# 吉西他滨联合长春瑞滨方案治疗复治中国晚期非小细胞肺癌患者的多中心回顾性研究

**DOI:** 10.3779/j.issn.1009-3419.2012.09.02

**Published:** 2012-09-20

**Authors:** 舜 陆, 力 张, 永峰 虞, 公琰 陈, 青 赵

**Affiliations:** 1 200030 上海，上海交通大学附属胸科医院肺部肿瘤临床医学中心 Shanghai Lung Cancer Center, Chest Hospital Affiliated to Shanghai Jiaotong University, Shanghai 200030, China; 2 510060 广州，中山大学肿瘤医院肿瘤科 Department of Medical Oncology, Cancer Center of Sun Yat-Sen University, Guangzhou 510060, China; 3 150081 哈尔滨，哈尔滨医科大学附属肿瘤医院 Department of Medical Oncology, the Third Affiliated Hospital of Harbin Medical University, Harbin 150081, China; 4 650032 昆明，昆明军区总医院胸外科 Department of Thoracic Surgery, Kunming Military General Hospital, Kunming 650032, China

**Keywords:** 吉西他滨, 长春瑞滨, 化疗, 肺肿瘤, Gemcitabine, Vinorelbine, Chemotherapy, Lung neoplasms

## Abstract

**背景与目的:**

本研究旨在回顾性分析使用吉西他滨+长春瑞滨（GN）方案治疗复治晚期非小细胞肺癌的疗效和安全性。

**方法:**

通过非干预的方式收集国内4家医院2004年1月1日-2010年6月30日间行GN方案治疗的晚期非小细胞肺癌二线或二线以上的患者，评价该化疗方案的疗效、无进展生存期、中位生存期和毒副反应。卡方检验比较二线和二线以上患者的疗效差异，应用*Kaplan-Meier*法进行生存比较和分析。

**结果:**

共53例患者在二线或二线以上采用了GN方案，其中二线患者28例，三线或三线以后的患者25例，所以患者均可评价疗效和不良反应。客观缓解率（objective response rate, ORR）为9.4%，疾病控制率（disease control rate, DCR）为56.6%，中位无进展生存期（progression free survival, PFS）为3.0个月，中位总生存期为17.6个月，多数患者的毒副反应可以耐受。单因素分析显示体能状况评分（performance status, PS）是影响患者PFS的因素。

**结论:**

吉西他滨联合长春瑞滨方案在晚期非小细胞肺癌复治患者中有较好的疗效和安全性，可以作为一种治疗选择。

## Abstract (en)

**Background and objective:**

The aim of this study is to detect the efficacy and toxicity of gemcitabine and vinorelbine (GN as second-line or further-line treatment) in refractory advanced non-small cell lung cancer (NSCLC) patients in China.

**Methods:**

We retrospectively reviewed 53 NSCLC patients treated with this agent at four hospital in China from Jan 01, 2004 to Jun 30, 2010. Survival analysis was evaluated by *Kaplan-Meier* method.

**Results:**

A total of 53 patients were analyzed in this study (28 patients in second-line and 25 in third- or further-line treatment). The objective response rate (ORR) was 9.4%. The disease control rate (DCR) was 56.6%. The progression-free survival (PFS) and median overall survival (OS) was 3.0 and 17.6 months, respectively. Univariate analysis, revealed performance status (PS) score was an independent prognostic factor for overall survival.

**Conclusion:**

The GN agent is effective for the second-line or further- line treatment in advanced non-small cell lung cancer. The toxicity is well tolerated.

肺癌是当前最常见的恶性肿瘤，其发病率和死亡率己占癌症之首。其中40%-50%的患者确诊时为晚期^[1]^。化疗是目前晚期非小细胞肺癌（non-small cell lung cancer, NSCLC）的标准方案，以铂类为主的联合方案（吉西他滨、紫杉醇、多西紫杉醇、长春瑞滨）^[2, 3]^化疗能明显延长患者生存期，改善患者生存质量，但有效率仅为30%-40%，中位无进展生存期仅为5个月左右，超过一半的患者在疾病进展后进入后续治疗。目前对于复治晚期非小细胞肺癌二线治疗，TAX317^[4]^、TAX320^[5]^以及JMEI^[6]^研究奠定了单药多西他赛和培美曲塞作为二线治疗化疗的标准地位。但目前对于二线单药化疗治疗其疗效有限，中位无进展生存期集中在3个月左右。对于一般状况许可的患者，两药方案也是复治患者常用的选择。对于既往治疗中没有使用过吉西他滨和长春瑞滨的患者，这两种药物的组合因为避免了含铂方案的肝肾功能及胃肠道损害，成为了一种可行的方案。

本研究回顾性收集国内多家医院既往采用吉西他滨联合长春瑞滨（GN）方案治疗复治晚期非小细胞肺癌患者的相关数据，旨在探讨国内GN方案在晚期非小细胞肺癌复治患者中的疗效及安全性，为临床诊疗提供参考。

## 1 资料与方法

### 1.1 临床资料

收集2004年1月1日-2010年6月30日在上海胸科医院、中山大学肿瘤医院、哈尔滨医科大学附属肿瘤医院及昆明军区总医院就诊的具有完整随访资料的Ⅲb期-Ⅳ期晚期NSCLC患者，共有120例患者接受了GN方案治疗，其中二线及二线以上患者中共53例患者使用了GN方案，28例患者在二线中采用了GN方案，25例患者在三线或三线以上采用了GN方案。患者一般特征及二线治疗和二线以后的患者的一般特征比较见表 1。

**1:** 53例患者的一般特征 Demographic characteristics of the study population

	Total (*n*=53)	Second-line (*n*=28)	Third- or further-line (*n*=25)	*P*
Gender				0.77
Male	35	19	16	
Female	18	9	9	
Age				0.79
Range	33-79	41-78	33-72	
Median	62	62	61	
< 65	37	20	17	
≥65	16	8	8	
Staging				0.51
Ⅲb	7	5	2	
Ⅳ	46	23	23	
PS				0.84
0-1	43	23	20	
2	10	5	5	
Histology				0.32
Adenocarcinoma	39	19	20	
Non-adenocarcinoma	14	9	5	
Smoking history				0.86
Yes	24	13	11	
No	29	15	14	
PS: performance status				

### 1.2 治疗方法

使用吉西他滨（商品名：泽菲）+长春瑞滨（商品名：盖诺）（江苏豪森药业生产）联合方案化疗。泽菲1, 000 mg/m^2^静脉滴注30 min，盖诺25mg/m^2^静脉滴注15 min-20 min。每21 d-28 d为1个周期。两组均不使用预防性升白细胞治疗。治疗期间根据需要使用粒细胞集落刺激因子、白介素11、止吐、抗感染等对症支持治疗。

### 1.3 疗效及毒性反应评定标准

根据实体瘤疗效评价标准（Response Evaluation Criteria in Solid Tumors, RECIST）1.1评价近期疗效，分为完全缓解（complete response, CR）、部分缓解（partial response, PR）、疾病稳定（stable disease, SD）和疾病进展（progressive disease, PD）。客观缓解率（objective response rate, ORR）=（CR+PR）/（CR+PR+SD+PD）×100%。疾病控制率（disease control rate, DCR）=（CR+PR+SD）/（CR+PR+SD+PD）×100%。根据美国国立癌症研究院通用毒性标准（Common Toxicity Criteria, CTC）评价不良反应（0度-4度）。

### 1.4 随访和生存分析

随访由各家医院采用门诊或电话方式，末次随访时间为2011年9月1日。总生存期（overall survival, OS）定义为患者自首次治疗起至患者死亡或末次随访的时间。无进展生存期（progression free survival, PFS）定义为患者自GN方案治疗开始至明确为疾病进展的时间。

### 1.5 统计学分析

应用SPSS 17.0软件进行统计学分析。计数资料的比较采用χ^2^检验，PFS和OS的分析采用*Kaplan-Meier*法。*P* < 0.05为差异有统计学意义。

## 2 结果

### 2.1 近期疗效

53例患者均可评价疗效，所有患者均完成至少1个周期的治疗，中位治疗周期数为3，其中无CR患者，PR 5例，SD 27例，PD 21例，总ORR为9.4%，DCR为56.6%。二线ORR为10.7%，DCR为57.1%，三线或三线以上ORR为7.1%，DCR为52%。无论ORR还是DCR均无统计学差异（*P*分别为0.89和0.71），具体疗效见表 2。

**2:** GN方案在晚期NSCLC的二线及二线以上治疗的短期疗效比较 Short-term efficacy of GN regimen as second-line or further-line therapy for patients with advanced NSCLC

Best response	Second-line (*n*=28)	Third-line (*n*=25)
CR	0	0
PR	3	2
SD	13	11
PD	12	12
ORR	10.7%	7.1%
DCR	57.1%	52.0%
CR: complete response; PR: partial response; SD: stable disease; PD: progressive disease; ORR: objective response rate; DCR: disease control rate; NSCLC: non-small cell lung cancer.		

### 2.2 PFS和OS

中位随访时间为24.5个月。53例患者获得了完整的PFS时间，52例患者获得了OS时间，其中1例患者失访。所有患者的中位PFS为3.0个月，52例完整随访的患者的中位OS为17.6个月，见图 1和图 2。

**1:**
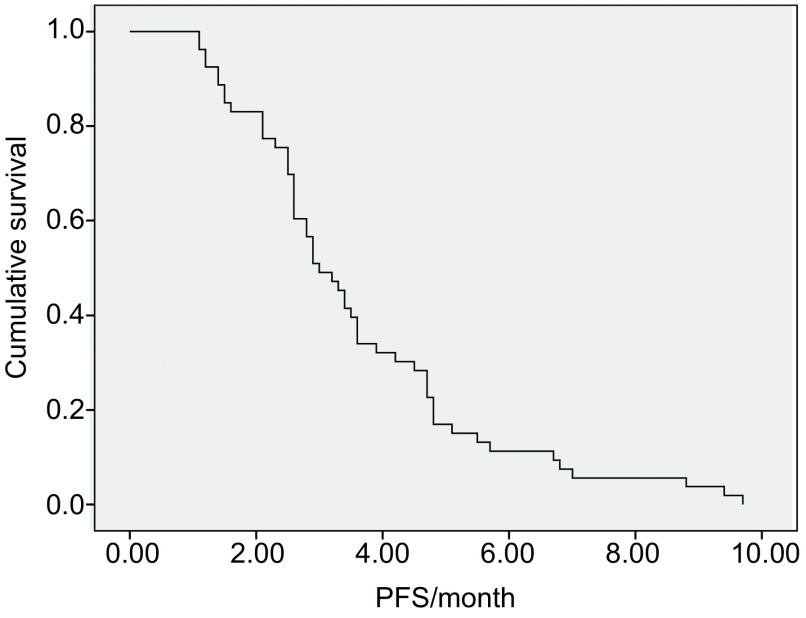
53例复治晚期非小细胞肺癌患者GN方案的PFS The PFS curves of 53 patients received GN regimen therapy.

**2:**
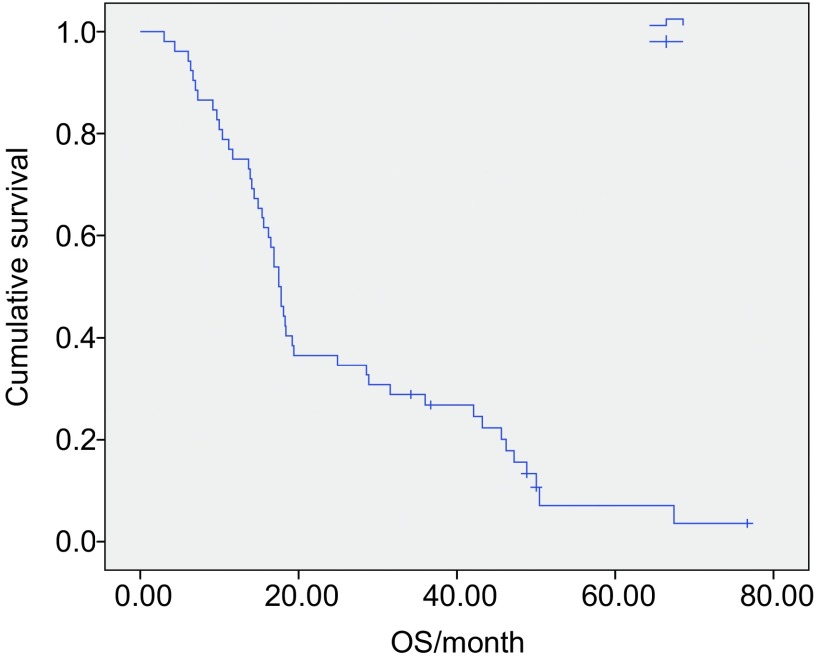
52例复治晚期非小细胞肺癌患者GN方案的OS The OS curves of 52 patients received GN regimen therapy. OS: overall survival.

### 2.3 影响患者PFS的单因素分析

无论患者性别、年龄、分期、病理类型、吸烟状况等均显示PFS无明显统计差异，但PS评分为0分-1分的患者PFS明显好于PS评分为2分的患者及（*P* < 0.01）（表 3，图 3，图 4）。

**3:** 53例患者PFS单因素分析 The PFS of the subgroup in the 53 patients

	*n*	PFS (month)	Range (month)	*P*
Gender				0.258
Male	35	3.0	2.2-3.8	
Female	18	2.8	1.9-3.6	
Age				0.534
Range	32-78			
Median	60			
< 65	37	3.2	2.6-3.8	
≥65	16	2.9	2.1-3.7	
Stage				0.190
Ⅲb	7	3.4	2.4-4.4	
Ⅳ	46	2.9	2.1-3.7	
PS				< 0.010
0-1	43	3.4	2.9-3.7	
2	10	1.4	1.1-1.7	
Histology				0.690
Adenocarcinoma	39	2.9	2.3-3.5	
Non-adenocarcinoma	14	3.0	1.4-4.7	
Smoking history				0.740
Yes	24	3.0	2.4-3.6	
No	29	2.8	1.7-3.9	
Which line				0.447
Second-line	28	3.4	2.6-4.2	
Third- or further-line	25	2.8	2.3-3.3	
PFS: progression free survival				

**3:**
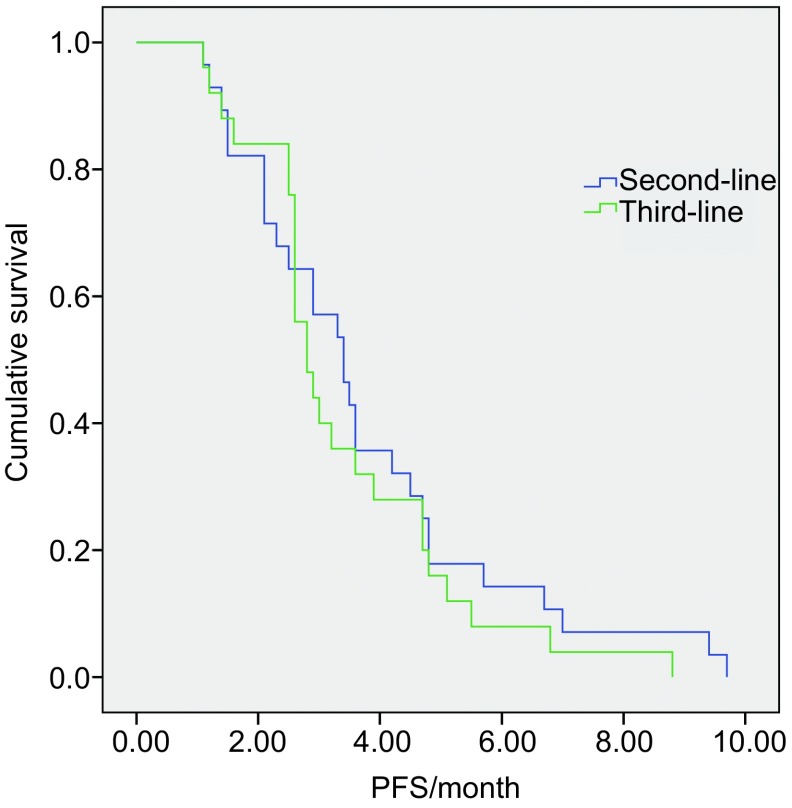
GN方案化疗在二线和二线以上复治患者中的PFS比较 Comparison of PFS between second-line and further-line with GN treatment (*P*=0.447)

**4:**
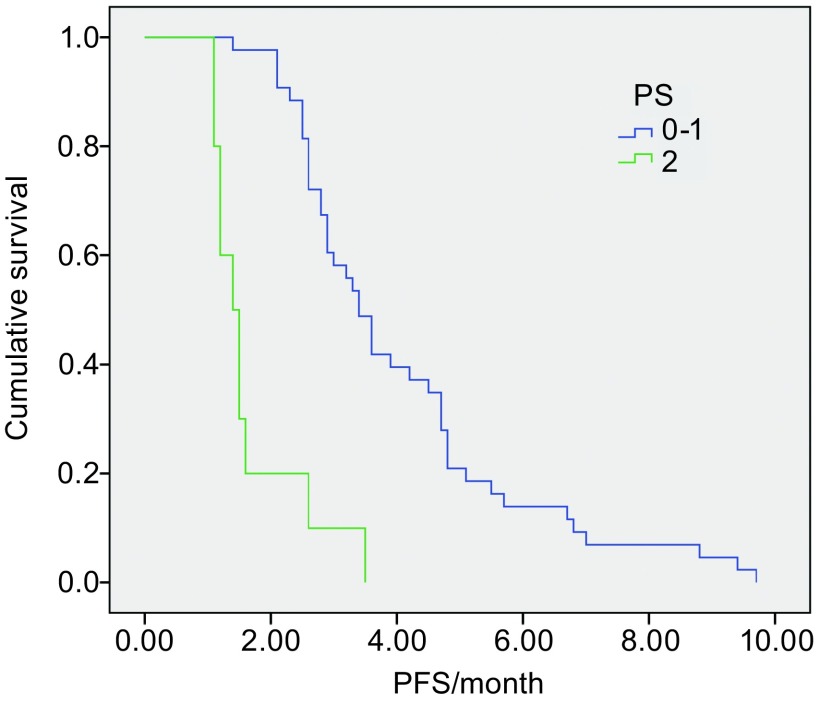
GN方案化疗的患者中不同PS评分患者的PFS比较 Comparison of PFS between different performance status (*P* < 0.01)

### 2.4 不良反应

多数患者的毒副反应为Ⅰ度-Ⅱ度，发生Ⅲ度-Ⅳ度不良反应的患者比例较低，其中厌食、静脉炎和恶心呕吐发生比例相对较高。未发生因毒副反应停药的患者。血液学毒性方面，中性粒细胞降低较为常见，其中Ⅰ度-Ⅱ度发生比例为37.7%（20/53），16例（30.1%）患者发生了Ⅰ度-Ⅱ度血小板降低。其它不良反应均在可控制范围内（表 4）。

**4:** 患者主要不良反应 The main toxicity of all the patients

Toxicity	Garde Ⅰ-Ⅱ toxicity	Garde Ⅲ-Ⅳ toxicity
Nausea/Vomiting	16	3
Anorexia	20	4
Alopecia	19	8
Dyspnoea	9	4
Diarrhoea	5	2
Neurotoxicity	2	1
Cough	6	3
Constipation	13	6
Pyrexia	9	2
Stomatitis	15	4
Myalgia	9	3
Thrombocytopenia	0	5
Neutropenia	20	10
Anaemia	13	4
ALT/AST	16	5
Phlebitis	21	4

## 3 讨论

本研究回顾性分析国内多家医院的GN方案治疗复治晚期非小细胞肺癌患者疗效显示，其客观有效率达到9.4%，疾病控制率达56.6%，中位无进展生存期为3.0个月，且毒副反应相对较轻，与目前复治晚期非小细胞肺癌标准药物疗效相当。

TAX317^[4]^和TAX320^[5]^两项大型多中心试验表明多西他赛在复治晚期NSCLC患者中有效率约为7%左右。JMEI^[6]^试验纳入571例既往不含培美曲赛或多西紫杉醇的一线化疗失败、ECOG评分0分-2分的晚期NSCLC患者，结果表明培美曲塞有效率达到9.1%，亚组分析表明培美曲塞在腺癌方面的有效性高于鳞癌。INTEREST^[7]^和BR21^[8]^及TRUST^[9]^试验则奠定了吉非替尼和厄洛替尼在二线治疗中的地位。二线单药治疗在有效率方面已经达到了一个治疗平台，总有效率低于10%。中位无进展生存期约为2个月-4个月。2009年的一篇荟萃分析^[10]^纳入6项研究，共847例患者，该荟萃分析评价了晚期非小细胞肺癌中二线治疗单药与双药的疗效，结果表明，双药治疗提高了患者的无进展生存期。该研究提示，对于部分体能状况较好的患者，双药化疗仍能给患者带来一定的获益。对于既往一线治疗中采用紫杉醇或多西紫杉醇方案的患者，在二线治疗中可能不会继续使用多西他赛。而培美曲塞和靶向药物有一定的病理类型的选择性，因此GN方案可能成为一种复治患者的治疗选择。Chen等^[11]^对7项临床试验中的数据分析比较显示，多西紫杉醇+异环磷酰胺，多西紫杉醇+吉西他滨，吉西他滨+长春瑞滨，长春瑞滨+卡铂在复治患者中有效率分别为10%、36.1%、31.3%、9.5%，中位进展时间分别为5.0个月、3.8个月、4.6个月、3.7个月。Kosmas等^[12]^进行的一项Ⅱ期临床试验评价了在既往紫杉醇联合铂类治疗失败后采用GN方案的疗效，共入组40例患者，结果有效率达到22.5%，DCR为55%，中位无进展生存期为4.5个月，不良反应在可耐受的范围内，提示GN方案在复治患者中有一定的疗效。本研究通过53例患者的分析表明，GN方案组合疗效可以接受，且毒副反应可以耐受。

作为一项回顾性分析，患者时间跨度较大，入组的样本量也较少，入组患者标准也不完全统一，因此评价GN方案对复治患者的疗效有一定的局限性。未来可以进行相关前瞻性研究进一步明确GN方案在疗效方面的作用。

本研究基于国内多中心的回顾性研究显示，在晚期非小细胞肺癌复治患者治疗中采用GN方案疗效和安全性均较好，可以作为一种较好的治疗选择。
